# Comprehensive Proteomic Profiling of Wheat Gluten Using a Combination of Data-Independent and Data-Dependent Acquisition

**DOI:** 10.3389/fpls.2016.02020

**Published:** 2017-01-10

**Authors:** Sophie N. L. Bromilow, Lee A. Gethings, James I. Langridge, Peter R. Shewry, Michael Buckley, Michael J. Bromley, E. N. Clare Mills

**Affiliations:** ^1^Faculty of Biology, Medicine and Health, Infection, Immunity and Respiratory Medicine, Manchester Academic Health Sciences Centre, Manchester Institute of Biotechnology, University of ManchesterManchester, UK; ^2^Waters CorporationWilmslow, UK; ^3^Rothamsted ResearchHarpenden, UK; ^4^School of Chemistry, Manchester Institute of Biotechnology, University of ManchesterManchester, UK; ^5^Synergy HealthHebden Bridge, UK

**Keywords:** proteomics, plant proteomics, mass spectrometry, chymotrypsin, QTOF-MS/MS, LTQ-Orbitrap, gluten, coeliac disease

## Abstract

Wheat is the most important food crop in the world, the unique physiochemical properties of wheat gluten enabling a diverse range of food products to be manufactured. However, genetic and environmental factors affect the technological properties of gluten in unpredictable ways. Although newer proteomic methods have the potential to offer much greater levels of information, it is the older gel-based methods that remain most commonly used to identify compositional differences responsible for the variation in gluten functionality, in part due to the nature of their primary sequences. A combination of platforms were investigated for comprehensive gluten profiling: a QTOF with a data independent schema, which incorporated ion mobility (DIA-IM-MS) and a data dependent acquisition (DDA) workflow using a linear ion trap quadrupole (LTQ) instrument. In conjunction with a manually curated gluten sequence database a total of 2736 gluten peptides were identified with only 157 peptides identified by both platforms. These data showed 127 and 63 gluten protein accessions to be inferred with a minimum of one and three unique peptides respectively. Of the 63 rigorously identified proteins, 26 were gliadin species (4 ω-, 14 α-, and 8 γ-gliadins) and 37 glutenins (including 29 LMW glutenin and 8 HMW glutenins). Of the HMW glutenins, three were 1Dx type and five were 1Bx type illustrating the challenge of unambiguous identification of highly polymorphic proteins without cultivar specific gene sequences. The capacity of the platforms to sequence longer peptides was crucial to achieving the number of identifications, the combination of QTOF-LTQ technology being more important than extraction method to obtain a comprehensive profile. Widespread glutamine deamidation, a post-translational modification, was observed adding complexity to an already highly polymorphic mixture of proteins, with numerous insertions, deletions and substitutions. The data shown is the most comprehensive and detailed proteomic profile of gluten to date.

## Introduction

Wheat is arguably the most important grain in the world and forms a staple part of the modern diet (Shewry and Tatham, 2016), being present in many processed foods including breads, noodles, pasta, biscuits, cakes and sauces (Kamal et al., 2009; Gao et al., 2016). Its versatility as a food ingredient results from the unique physicochemical properties of the gluten fraction of wheat seed protein. One of the earliest proteins to be studied, gluten was first described by Beccari in 1728 (Bailey, 1941), and is readily isolated from wheat flour as a viscoelastic mass by making dough and then washing it with dilute salt solutions. Gluten comprises the major storage proteins of wheat grain, which are traditionally divided into two groups based on their solubility called gliadins and glutenins (Osborne, 1907). The gliadins comprise monomeric subunits which are soluble in alcohol-water mixtures and are further classified, based on their mobility on electrophoresis at low pH, into α-,γ-, and ω-gliadins. The glutenins comprise two groups of subunits, called high molecular weight (HMW) and low molecular weight (LMW) glutenin subunits (Bietz and Wall, 1973, 1980), which form alcohol-insoluble polymers stabilized by inter-chain disulfide bonds. However, when the disulfide bonds are reduced, the glutenin subunits become soluble in aqueous alcohol and amino acid sequences show that gliadins and glutenin subunits are related. A characteristic of both groups is that they contain few arginine and lysine residues and are rich in the amino acids proline and glutamine, which result from the presence of large domains comprising repetitive short peptide sequence motifs dominated by proline and glutamine. It is therefore usual to define both as prolamins, the name originally applied only to wheat gliadins and related proteins from other species.

Wheat is very diverse, with thousands of different types cultivated across the world, and over 40 cultivars currently being recommended for growth in the UK alone. The bread making quality of wheat is associated particularly with allelic variation in the HMW subunits of glutenin but these are not the sole determinants of quality. Many other factors are involved such as variation in gluten proteins other than HMW subunits, especially those that may modify polymer formation. However, these are generally poorly understood and make bread making quality of difficult to predict (Groger et al., 1997; Liu et al., 2016). Gel-based methods have long been used to identify variations in gluten protein composition associated with bread making quality (Dupont et al., 2011) whilst more recently methods employing MS alone in bottom-up approaches has been used for profiling gluten proteins (Mamone et al., 2011; Prandi et al., 2014; Colgrave et al., 2015; Manfredi et al., 2015; Wang et al., 2015; van den Broeck et al., 2015; Barro et al., 2016; Martínez-Esteso et al., 2016). One study using data independent acquisition (DIA) identified only a few gluten proteins as a consequence of employing only tryptic digestion (Uvackova et al., 2013). Data dependent acquisition (DDA) of classical Osborne fractions prepared from flour has also allowed identification of several hundred gluten peptides (Fiedler et al., 2014), whilst another study identified more than 80 wheat-specific proteins, including gluten, albumin and globulin proteins using a gluten-enriched fraction (Colgrave et al., 2015). A more recent analysis of a gluten food ingredient, coupled with fractionation to enrich gluten proteins also allowed identification of several hundred gluten-specific peptides (Martínez-Esteso et al., 2016). Due to the complexity of gluten, a large proportion of the current MS analysis carried out on gluten has been focused to profile HMW glutenin subunits initially (Lagrain et al., 2013; Wang et al., 2014).

Wheat variety Hereward, which is classified as a group 1 winter wheat, shows consistent baking and milling properties, and is well suited to bread making (Nabim Wheat Guide, 1978). However, this does not explain its good bread making quality as Hereward contains a combination of HMW subunits (called subunits Bx7+By9 and Dx3+Dy12) which are associated with poor quality (Shewry et al., 2012; Bekderok et al., 2013). Using Hereward as a model wheat we aim to comprehensively characterize the gluten proteome using a linear ion trap (LTQ) instrument in a DDA mode and a quadrupole time of flight (QTOF) instrument incorporating ion mobility in a DIA mode. A curated database containing only full length gluten protein sequences was used for annotation in order to overcome shortcomings of uncurated public repositories.

## Methods

### Plant materials and reagents

Grain of the bread making wheat (*Triticum aestivum)* cultivar Hereward was grown at Rothamsted Research, Harpenden. Total protein was determined by nitrogen analysis of wholemeal flour using the Dumas combustion method (Serrano et al., 2013) multiplied by a factor of 5.7 giving a mean of 13.5% protein by dry weight for three replicate 1 g samples.

All reagents used were analytical grade unless stated otherwise. Formic acid, acetonitrile and water used in chromatography were all HPLC grade (Sigma-Aldrich, Dorset, UK). α-Chymotrypsin (Merck Chemicals, Nottingham, UK) with an activity of ≥300 U/mg (measured by ATEE assay) and a specific activity 400 U/mg of protein was used for digestion of the gluten proteins. Rapigest™ (a patented surfactant used to enhance enzymatic digestion of proteins by helping solubilize proteins, making them more susceptible to enzymatic cleavage without inhibiting enzyme activity; Yu and Gilar, 2009) and Hi3 PhosB standard (Waters 186006011, Wilmslow UK) were provided by Waters Corporation, Manchester. ZipTips C_18_ (Sigma-Aldrich, Dorset, UK) with a volume of 0.1-10 μL were used for the as part of the mass spectrometry preparation step. NuPAGE Bis-tris gels (12%), NuPAGE lithium dodecyl sulfate (LDS) buffer (4X, pH 8.4) and SimplyBlue™ safestain were from Invitrogen (Shropshire, UK). Mark 12™ marker and SeeBlue™ prestained marker were also from Invitrogen. Secondary anti-mouse IgG labeled with alkaline phosphatase and nitro-blue tetrazolium chloride (NBT)/5-bromo-4-chloro-3'indolyphosphate p-toluidine salt (BCIP) substrate solution were sourced from ThermoScientific (Leicestershire, UK). Blotting membrane 0.2 μm pore size was sourced from BioRad, Hertfordshire, UK. Mouse monoclonal antibodies (mAbs) IFRN 0610 (toward QPFP epitope; Brett et al., 1999), G12 (anti-gliadin 33mer; Morón et al., 2008; were provided by Adrian Rogers, Romer Labs UK Ltd) and R5 (toward QXPFP, QQQFP, LQPFP; Valdés et al., 2003; Kahlenberg et al., 2006; Operon, Zaragoza, Spain) were used in immunoblot analysis.

### Experimental design and statistical rationale

A sample of seeds (*n* = 100 grain) was weighed after discarding any shriveled seeds and the average grain weight found to be 49.47 mg. Three plump seeds per extraction were selected to be representative and had a weight similar to the average weight for the total seed sample (±7.13 mg). Three seeds were extracted per type of buffer and each extract analyzed in triplicate by LC-MS using the QTOF (giving pooled data from 27 analyses from nine seeds) and in duplicate using the LTQ (giving pooled from 18 analyses from nine seeds). Three technical replicates of the MS acquisition were used to calculate the mean protein abundances and allow statistical analysis to be carried out in measurements. A summary of the experimental workflow can be found in Supplementary Material, Figure S1.

Controls implemented include the use of LeuEnk during the detector set up of the mass spectrometer and Hi3 PhosB standard (Waters, Wilmslow, UK) was used as a standard for all sample preparation and verifying instrument performance. Samples were randomized for analysis and blank injections of MilliQ water were carried out every three injections.

Variation between protein extractions was calculated as the standard deviation. Variation was also accessed on a run to run basis for all peptides residues to investigate the occurrence in the biological and technical replicates.

### Protein extraction

Individual wheat grains (cv Hereward) were crushed between filter paper using pliers, and transferred to a clean Eppendorf. To three crushed grains (per extraction) 250 μL of extraction buffer was added.

(E1) 50 mM Tris-HCl (pH 8.8), 50 mM DTT and (0.2% w/v) Rapigest™.
(E2) 50 mM Tris-HCl (pH 8.8), 50 mM DTT, (0.2% w/v) Rapigest™ and 75% (v/v) ethanol.
(E3) Two Step Sequential extraction: 50 mM Tris-HCl (pH 8.8), 50 mM DTT, Rapigest (0.2% w/v), with the pellet resuspended using 50 mM Tris-HCl (pH 8.8), 50 mM DTT, Rapigest (0.2% w/v), 75% ethanol (v/v).

Rapigest™ was included in all extracts at 0.2% (w/v) in order to improve protease digestion. All extractions were carried out for 15 min with sonication in a water bath heated to 60°C (VWR, Leicestershire, UK), vortexing every 5 min. Extracts were then centrifuged at 10,000 × g for 10 min, the supernatant collected and transferred to a clean microcentrifuge tube. Three biological replicates were extracted with each buffer. Protein concentration of the biological replicates for each extraction was initially determined using 2D Quant Assay™ (GE Healthcare, Buckinghamshire UK) as per the manufacturer's instructions using bovine serum albumin as a standard. The protein extraction rate was calculated as a percentage of the total grain protein determined with a Kjeldhal analysis using the Dumas combustion method for each extraction.

### SDS PAGE and immunoblots

One of each of the triplicate biological replicates for each extraction was prepared for SDS PAGE and immunoblot analysis as per (Smith et al., 2015).

#### Sample preparation for mass spectrometry

Samples extracted using buffers E1–E3 (25 μL) were reduced by addition of 330 μL of 50 mM ammonium bicarbonate and 40 μL of 50 mM DTT and heated to 80°C for 10 min. After allowing to cool to room temperature, 45 μL of 150 mM iodoacetamide was added and samples were incubated at room temperature in the dark for 30 min. A two-step digestion protocol was used as follows: (1) to each sample 25 μL of 0.1 mg/mL chymotrypsin was added and incubated in 37°C for 4 h; (2), 25 μL of 0.1 mg/mL of chymotrypsin was then added and the samples incubated overnight at 37°C. Each sample then underwent off-line desalting on C_18_ Ziptips and was diluted to 1 mg/mL protein using 0.2% (v/v) acetonitrile containing 0.1% (v/v) formic acid. Hi3 PhosB standard (Waters 186006011, Wilmslow, UK) was spiked into samples at 100 fmol and samples were loaded on column at 100 ng/μL.

Each sample was subject to liquid chromatography coupled with tandem mass spectrometry (LC-MS/MS) analysis on both QTOF (quadrupole time of flight) (Waters, Wilmslow, UK) and LTQ (linear ion trap quadrupole) (Thermo Fisher Scientific, Waltham, MA, USA) MS platforms. Only two of the three biological replicates were analyzed by LTQ.

QTOF (quadrupole time of flight): Aliquots (1 μL) of digested sample extracts E1-E3 were chromatographically separated on an M-class ACQUITY UPLC system (Waters, Wilmslow, UK) using a NanoEase 1.8 μm HSS T3 C18 (75 μm × 150 mm) column (Waters) using a linear gradient (flow rate 300 nL/min) from 3 to 40% (v/v) solvent B over 90 min. The mobile phases consisted of solvent A (0.1% (v/v) formic acid/99.9% (v/v) water) and solvent B (0.1% (v/v) formic acid/99.9% (v/v) acetonitrile). The eluate was directed into the electrospray ionization source of the Synapt G2-Si™ (Waters Corporation Wilmslow, U.K). Data was acquired in ion mobility assisted data independent analysis (IM DIA) mode. MS analysis was performed in positive ion mode over the mass range *m/z* 50–2000 with a 0.5 s spectral acquisition time. One cycle of low and elevated energy data was acquired every 1 s.(Rodriguez-Suarez et al., 2013)

LTQ: Data acquisition was carried out by the Biological Mass Spectrometry Facility of the University of Manchester. Aliquots (1 μL) of digested sample extracts E1-E3 were chromatographically separated using an UltiMate® 3000 Rapid Separation LC (RSLC, Dionex Corporation, Sunnyvale, CA) coupled to an Orbitrap Elite (Thermo Fisher Scientific, Waltham, MA, USA) mass spectrometer. Peptide mixtures were separated using a gradient from 92% (v/v) A (0.1%(v/v) formic acid/99.9% (v/v) water) and 8%(v/v) B (0.1%(v/v) formic acid/99.9%(v/v) acetonitrile) to 33% (v/v) B, in 44 min at 300 nL min^−1^, using a 75 mm × 250 μm, 1.7 Å M BEH C18, analytical column (Waters, Wilmslow UK). Data was acquired in the DDA mode. MS analysis was performed in positive ion mode over the mass range m/z 350–1500 with a 0.01 s spectral acquisition time. Peptides were selected for fragmentation automatically by data dependent analysis; +2 or +3 precursor ions and previously observed ions were excluded from fragmentation for a 30 s accumulation period.

#### Data processing

The LC-MS data from both platforms were processed using Progenesis QI for proteomics v2.0 (Li et al., 2009), and searched against a curated gluten database (Glu.Pro Ver1) that was based on well characterized sequences (Khan and Shewry, 2009). Searching of the two data sets were carried out differently due to the inherent differences in the data produced from the different instruments, LTQ data was searched using MASCOT whereas the QTOF gathered data was searched using PLGS. The database was curated from BLAST searching well annotated gluten sequences against the entire UniProt database, and downloading the resulting sequences. The resulting sequences were manually interrogated to remove sequences that were fragments, duplicates or assigned to a grass other than Triticum. The database comprises 634 unique full length cDNA sequences and was curated using Clustal Omega, DB-toolkit and Jalview (Martens et al., 2005; Waterhouse et al., 2009; McWilliam et al., 2013). Phylogenetic trees of the complete curated database were created using Figtree, and the Neighbor Joining BLOSUM62 algorithm (Rambaut, 2007). Carbamidomethylation of cysteine residues (+57.02 Da) was selected as a fixed modification, whilst oxidized methionine (+15.99 Da) and deamidated asparagine/glutamine (+0.984 Da) were selected as variable modifications. Chymotrypsin was the digestion enzyme of choice with the cleavage specificity set to tyrosine, tryptophan, phenylalanine and leucine unless preceded by a proline. Up to two missed cleavage sites were acceptable for chymotrypsin digestion. The false discovery rate (FDR) was set to 1%. Data from both platforms were handled identically to ensure data standardization and to allow for a robust comparison of mass spectrometry platforms. Mass tolerance for the precursor and fragments ions were set to 10 and 20 ppm respectively and the peptide threshold score set to 5 (Li et al., 2009). Quantification was done using top 3 protein quantification (T3PQ) (Silva et al., 2006) where for each protein identified by a set of unique peptides, the average of the three most efficiently ionized peptides correlates to the protein abundance observed. Uniqueness of peptides was considered using (1) only unmodified peptide sequences; and (2) peptide sequences with the inclusion of modifications resulting in different peptides of the same sequence. When deamidation was observed in multiple positions for one peptide sequence these were considered to be a single unique peptide as the exact location of the modification cannot be certain.

#### *In silico* digestion

*In silico digestion* was carried out on two HMW glutenin protein sequences (Uniprot accession P08489 and D0IQ05) attributed to two different HMW subunits to identify all theoretical peptides. The *in silico digestion* was carried out using ExPASy (http://www.expasy.org/) utilizing the Peptide Mass tool with cleavage rules of chymotrypsin (C-term to F/Y/W/M/L, not before P) set. The mass range was unlimited. The lists of resulting peptides for both proteins were downloaded and compared, and unique peptides for both proteins were identified.

## Results

**Figure 1:**
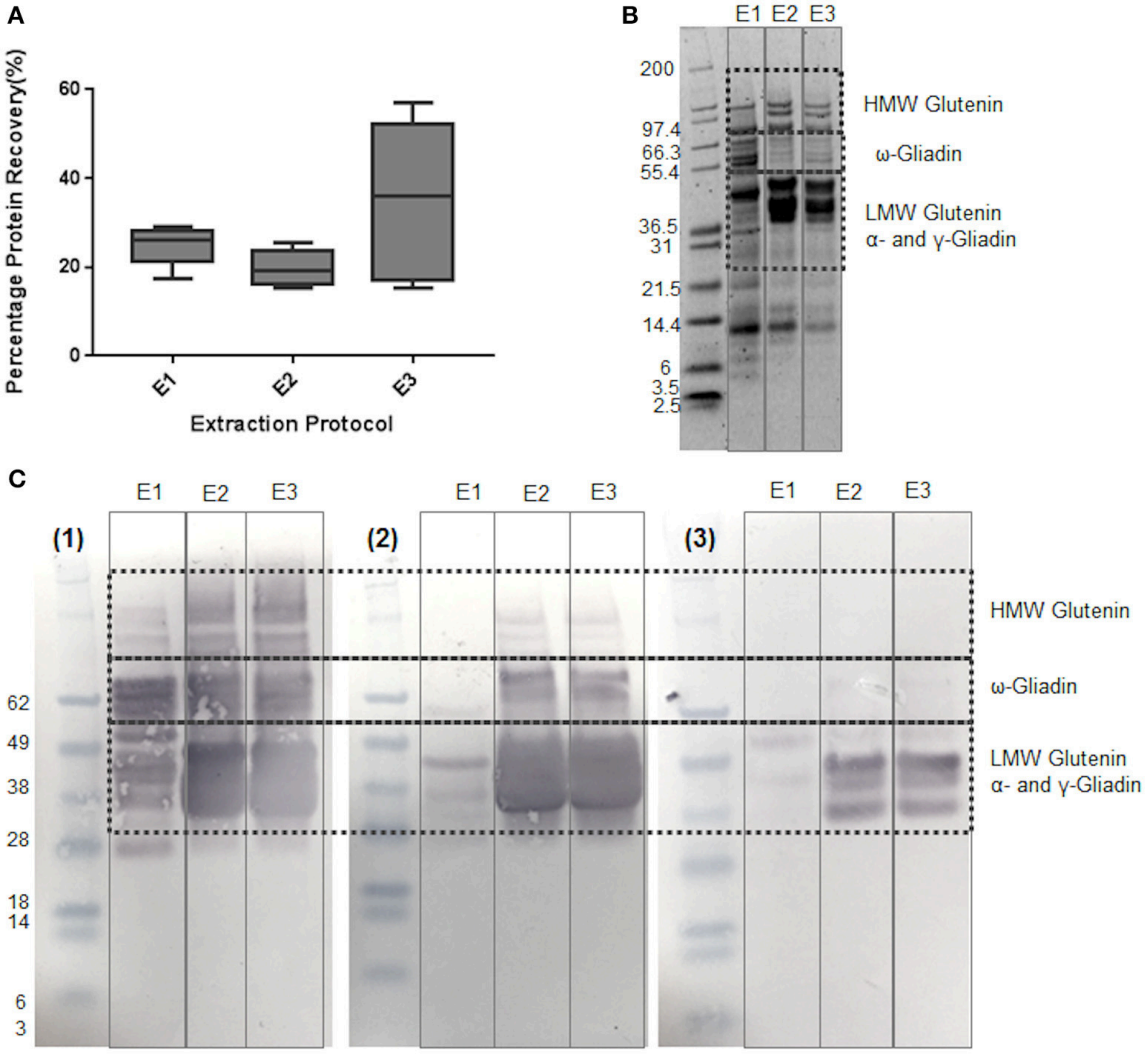
**Effect of different buffers on extraction of proteins from wheat grain c.v Hereward. (A)** Box and whisker plots of % protein recovery for buffers E1-E3. Percentage protein recovery was calculated as (protein extracted/protein present) x 100. Extraction solutions were as follows: (E1) 50 mM Tris-HCl (pH 8.8), 50 mM DTT, Rapigest (0.2% w/v), (E2) 50 mM Tris-HCl (pH 8.8), 50 mM DTT, Rapigest (0.2% w/v), 75% (v/v) ethanol; (E3) 2 Step extraction: 50 mM Tris-HCl (pH 8.8), 50 mM DTT, Rapigest (0.2% w/v), with the pellet resuspended using 50 mM Tris-HCl (pH 8.8), 50 mM DTT, Rapigest (0.2% w/v), 75% (v/v) ethanol. **(B)** SDS-PAGE analysis of protein extracts. Samples were prepared with buffers E1, E2, and E3 using one of the three biological replicates used for subsequent analysis. Mark 12 prestained molecular weight markers were used, with key gluten protein bands highlighted. **(C)** Immunoblotting analysis of protein extracts prepared with buffers E1, E2, and E3. Immunoblots were developed using anti-gluten antibodies as follows: (1) Mab IFRN 0610; (2) R5, and (3) G12 Molecular weight markers were prestained Seeblue markers. Key gluten protein bands are highlighted.

The effectiveness of different types of buffer on the extraction and detection of gluten proteins was initially assessed using several gluten-specific antibody preparations in order to identify a simple single step extraction method that was effective and compatible with subsequent MS workflows. Buffer E1, previously used for extraction of peanut proteins (Johnson et al., 2016) which primarily extracts the water-soluble (albumin) and salt-soluble (globulin) proteins of wheat, recovered 24.9% protein from the grain (Figure 1A) and revealed a complex mixture of polypeptides of 6–116 kDa (Figure 1B). Although immunoblotting with the mAb IFRN 0610 indicated that some gliadin and glutenin polypeptides had been extracted, only weakly reactive polypeptides were observed with the G12 and R5 antibodies (Figure 1C). In order to improve the extractability of the gliadin and glutenin fraction 75% (v/v) ethanol was added to buffer E1 to give buffer E2. It slightly reduced the extent of protein extraction to 19.8% but SDS-PAGE analysis revealed a pattern of polypeptides much more consistent with the pattern observed for gliadins and glutenins with the HMW subunits of cv Hereward (7 + 9, 3 + 12) being clearly visible. The E2 extract was strongly reactive with all three antibodies, although more polypeptides were recognized by the Mab IFRN0610 than either the R5 or G12 antibodies. This might be expected since the IFRN 0610 epitopes are present in many different seed storage prolamins from wheat (Brett et al., 1999; Kahlenberg et al., 2006; Mokarizadeh et al., 2015). Thus, the inclusion of reducing agent and ethanol improved the solubilization of gliadins and glutenins.

The two-step extraction which involved extraction of the albumin and globulins prior to that of the gliadins and glutenins gave a slightly higher rate of overall protein extraction but the variation in extraction efficiency between samples was increased (Figure 1A). This is probably due to the difficulties in undertaking the two-step extraction rather than inherent seed-to-seed variation in protein content because seeds of a similar size and therefore protein content were used for all extractions. This two-step extraction approach yielded a pattern of polypeptides following SDS-PAGE analysis very similar to that of the buffer E2 extract and showed the same pattern of reactivity with the different antibodies (Figure 1C).

**Figure 2:**
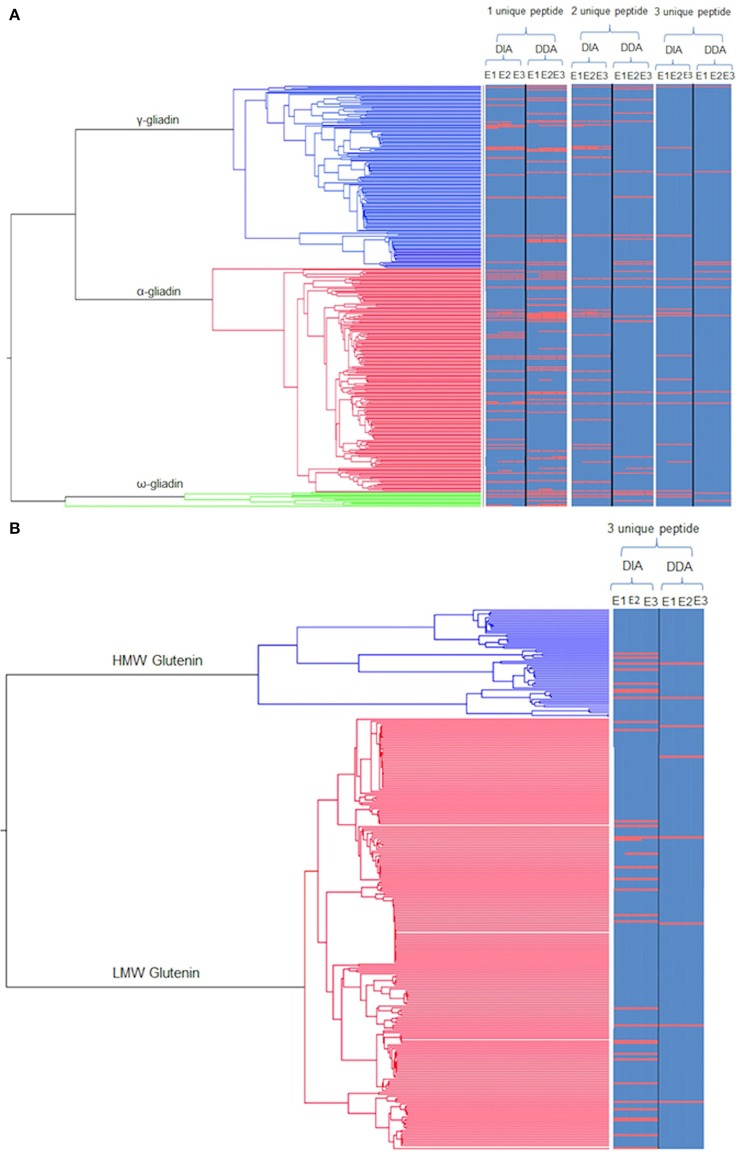
**Phylogenetic trees of gluten protein sequences mapped with identifications from the different extracts using the different platforms. (A)** Phylogenetic tree of the monomeric gliadins (α-gliadin: red, γ-gliadins: blue; and ω-gliadins: green) linked to a heat map showing the protein identifications as a red line for each extract (E1–3) as a function of the minimum number of unique peptides per protein across the two modes of acquisition (DIA, DDA). Protein accessions identified by DIA and DDA are indicated denoted by a continuous red line, with an off-set red line indicating closely related, but distinct isoforms, identified by only one MS platform. **(B)** Phylogenetic tree of the polymer (glutenin) gluten proteins (HMW: Blue and LMW: Red) linked to a heat map as for **(A)** but only showing the protein identifications made using a minimum three unique peptides per protein.

**Figure 3:**
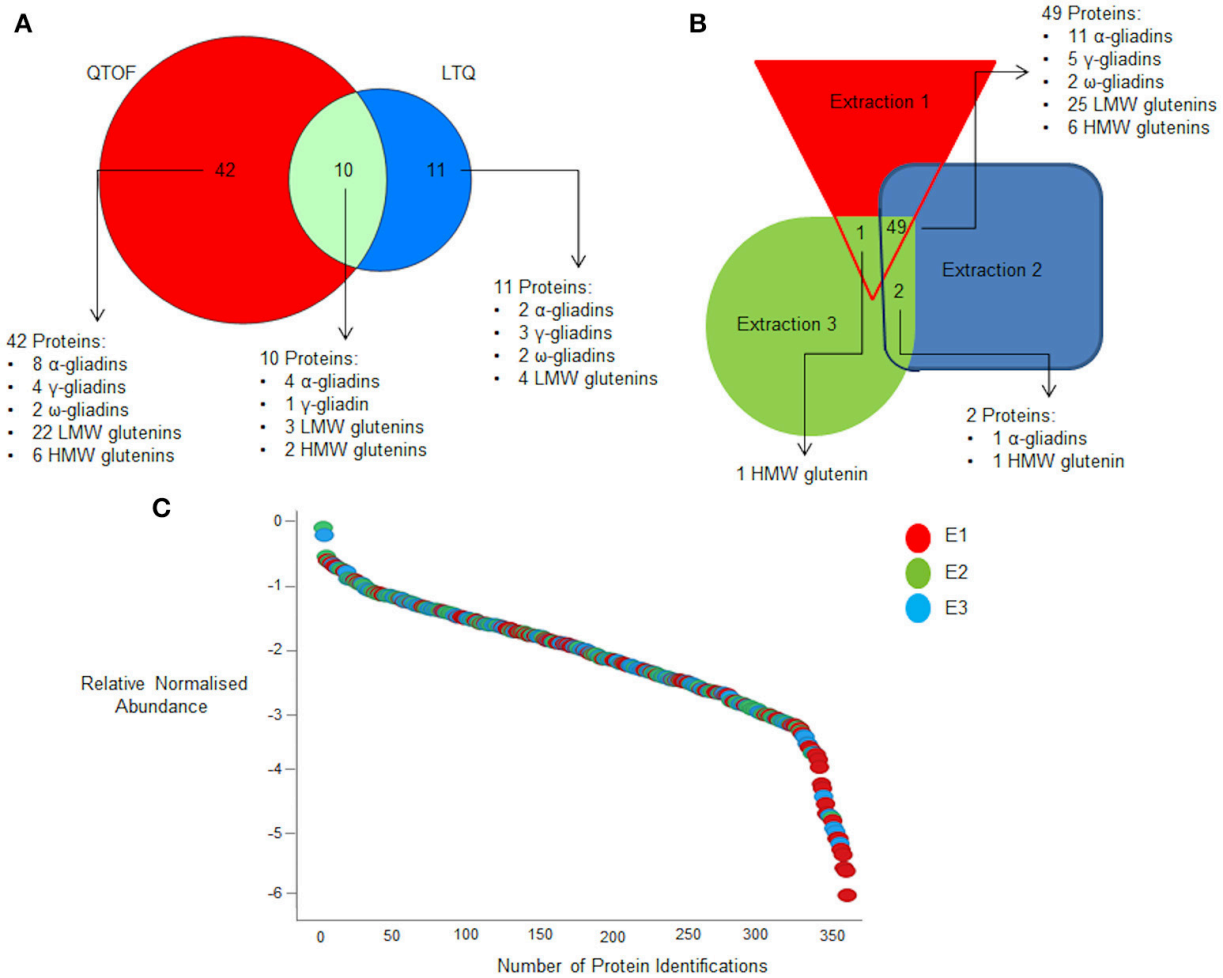
**Effect of extraction buffer on gluten protein identifications. (A)** Summary of the total number of proteins identified for all three extractions that are common and unique to the two mass spectrometry platforms when a criteria of three unique peptides is set. **(B)** Effect of extraction conditions platform on protein identification on QTOF when a criteria of three unique peptides is set. **(C)** Quantification curve of all proteins identified across the three extractions with E1, E2, and E3 being shown in red, green and blue respectively. Data are summarized in Data Sheet 1.

The same extracts were then subjected to LC-MS analysis using two different platform technologies operating in different acquisition modes. The LTQ is a linear trap quadrupole made up of two mass analyser the first being an ion trap that acts as a mass filter, followed by a quadrupole were the filtered ions are separated according to the *m/z* as they pass along the central axis of four parallel rods in an electrostatic field. Whereas the QTOF incorporated ion mobility separates the ions as described previously (Allen et al., 2016) followed by additional separation in the time of flight tube that accelerates ions by an electric field, and the time taken to travel a known distance is recorded. The ion mobility adds an additional level of separation of the ions in the gas phase based on the shape and charge of the ions, (Allen et al., 2016) The LTQ was operated in DDA mode of acquisition, where MS/MS scans are only acquired on a subset of precursors detected in an MS “survey” scan adhering to a minimum threshold, and is biased to pick peptides of the highest intensity (Egertson et al., 2015). The QTOF was operated using a DIA mode of acquisition whereby all peptides are fragmented and MS/MS scans collected regardless of signal intensity (Doerr, 2015). The resulting spectral libraries were analyzed using an in-house curated wheat gluten protein sequence database. This allowed a total of 2,736 gluten peptides to be identified across all the extracts analyzed, of which 1548 were identified by the QTOF and 1031 by the LTQ, with only 157 gluten peptides matched by both platforms. Subsequently these data were used to identify proteins on the basis that the same unique peptide(s) were observed in at least two replicate analyses of each extract from at least two seeds. Individual peptide scores ranged from 5 to 70.78 showing the confidence in the protein identifications (Supplementary Data Sheets 2, 3). A total of 127 protein accessions were identified on the basis of a single unique peptide, which was decreased to 63 if three unique peptides were required for a positive identification (Figures 2A, 3A), a list of the resulting 63 protein accessions with corresponding number of unique peptides and normalized relative abundance can be found (Supplementary Data Sheet 1). A phylogenetic tree of the gliadin (Figure 2A) and glutenin (Figure 2B) sequences from the database was used to visualize the protein identifications inferred from analysis of all the different extracts (E1–3) as a function of platform technology and, for the gliadins only, the number of unique peptides. Some protein accessions were identified by both mass spectrometry platforms and sometimes closely related, but distinct isoforms, were identified by different platforms. When three unique peptides were required to make an identification, a total of 19 gliadin species were identified which were distributed between the two main branches of the gliadins. Thus, four of the 12 ω-gliadin sequences were identified on one branch and 22 (14 α-gliadin and eight γ-gliadin species) out of a total of 340 α-gliadin and γ-gliadin protein sequences from the second. The LMW subunits form the largest class of glutenin sequences in the database, with 224 accessions, the HMW subunits being less abundant with only 57 sequences (Figure 2B). A total of 29 LMW subunit and eight HMW subunit sequences were identified, the majority of which were identified only by the QTOF platform. A total of 29 LMW glutenin and eight HMW glutenin sequences were identified, the majority of which were made only by the QTOF platform. Even though extract E1 contained little immunoreactive gluten (Figure 1) the majority (94%) of gluten proteins identified with at least three unique peptides were observed in all three extracts (E1–E3; Figures 2A,B, 3B; Data Sheets 1–4) with the majority of proteins identified in E1 having a lower relative normalized abundance compared to proteins identified in the two other extractions (Figure 3C). Differences in identifications made between extracts were limited to one additional LMW subunit of glutenin present in extracts E1 and E3 with a further LMW subunit of glutenin and an α-gliadin being identified in extracts E2 and E3 (Figure 3B). The QTOF and LTQ showed >75% and >82% commonality for all peptides identified across all biological and technical replicates with each extraction.

Since cv Hereward is known to contain only four HMW subunits (Dx3 + Dy12 and Bx7 + By9) (Belderok et al., 2000; Cunsolo et al., 2004) the identification of eight (three unique peptides criteria) and 12 (one unique peptide criteria) HMW subunit accessions was unexpected. These identifications were further investigated by inspecting the sequence identity for the available relevant HMW subunit accessions. Subunit Dx3, which is a rare allele and is yet to be sequenced. However, it usually considered to be closely related to HMW subunit Dx2, having a very similar molecular mass determined by ESI-MS (Bellil et al., 2012) and being associated with the Dy12 allele. Dx2 was therefore used as a surrogate for Dx3. A table of the eight identified HMW glutenin sequences are shown in Supplementary Material, Table S1. The eight identified sequences were compared to the two expected HMW subunit sequences (Uniprot accession P08489 and Uniprot accession Q42451) using sequence identity calculated using Clustal Omega (McWilliam et al., 2013). The sequences comprised two sequences closely related to Dx2 (Uniprot accession P08489) and five to By7 (Uniprot accession Q42451) with respective average percentage sequence identities of 81.5 and 95.3% (Supplementary Material, Table S1). The eight identified HMW glutenin subunits identified using a minimum of 3 unique peptide criteria were mapped onto a phylogenetic tree of the HMW glutenins to show the homologous relationship between the observed proteins (Supplementary Material, Figure S2). When the closely related sequences were further interrogated it was shown that these sequences belonged to subunits not expected to be present in wheat cv Hereward. An example of this is protein D0IQ05, a well annotated Dx5 sequence that shows a sequence identity to Dx2 subunit (Uniprot accession P08489) of 94%. Following *in silico* chymotryptic digestion of the two proteins corresponding to the Dx2 and Dx5 subunits (Uniprot accession P08489 and Uniprot accession D0IQ05) the 16 and 11 unique peptides were mapped onto the protein sequence (Supplementary Material, Figure S2). This shows unique peptides with either single amino acid mutations shown in blue or insert regions shown in purple for both proteins. The list of theoretical unique peptides for both proteins were searched through the global proteomics data, and of the 16 and 11 unique peptides for the Dx2 and Dx5 subunits nine and seven were identified in the MS data. The first unique peptides identified in both proteins after the start of the repetitive domain (as indicted by an arrow, Figure S3) varied by only one amino acid and were indicative of the different HMW subunits, with the serine present in the Dx2 protein being replaced by a cysteine in the Dx5 proteins. This change is a result of a single change in the second base of the genetic code. These two unique characteristic peptides were searched in the global proteomics data collected, and the partial MS/MS spectra for both are shown in Figures S4, S5 confirming the presence of both peptides and the identification of both HMW subunits. When the stringency requirements for identification were relaxed to a single unique peptide, the number of HMW subunits was increased to five homolog of Dx2 and seven homolog of Bx7, together with two homolog of Dy12 and three for By9. It seems likely that the identification of multiple homologs results from the close sequence similarity of the different isoforms and the fact that the HMW subunits of cv Hereward have yet to be sequenced and may have a combination of the single amino acid polymorphisms represented by the accessions identified.

**Figure 4:**
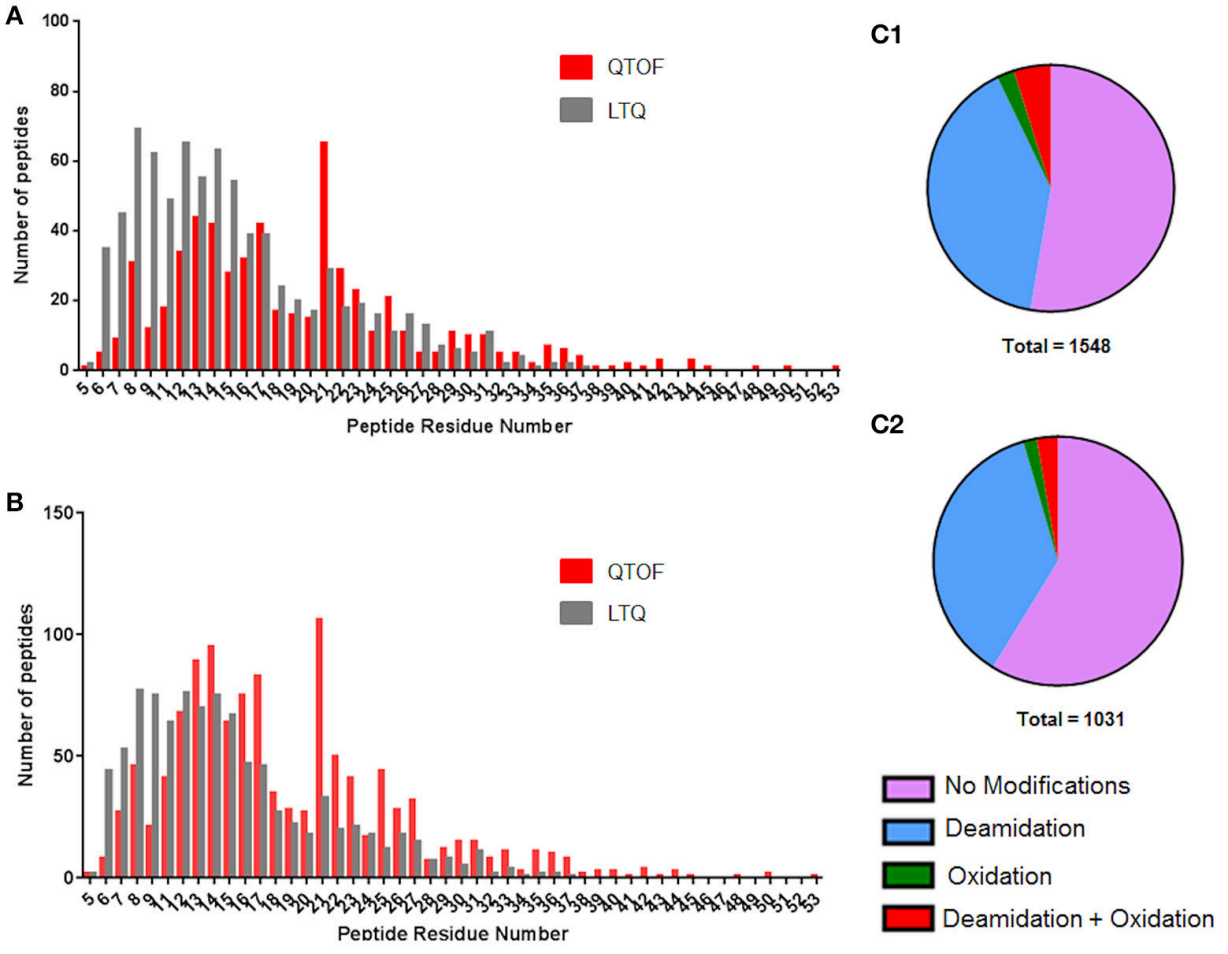
**Distributions of peptide lengths identified by LTQ and QTOF platforms as a function of residue modifications. (A)** Peptide length distribution of unique peptides when modifications are discounted for the QTOF (red) and LTQ (gray). **(B)** Peptide length distribution of unique peptides when modifications are taken into account for the QTOF (red) and LTQ (gray). **(C)** The distribution of modified and non-modified peptides identified by the **(C1)** QTOF and **(C2)** LTQ platforms. Data are summarized in Data Sheets 2 and 3.

Long peptides were identified by both platforms; the longest identified by the LTQ being 33 residues in length whilst the QTOF identified one peptide 53 residues in length, an exemplar partial MS/MS spectra for a 50 residue peptide identified using the QTOF is shown in Figure S7. None of the 33 residue peptides identified corresponded to coeliac toxic 33-mer (Mokarizadeh et al., 2015) to which the G12 antibody was raised. This was expected as the 33-mer is derived from simulated gastrointestinal digestion and is not flanked by chymotryptic cleavage sites. Instead five of the 33mer peptides were from γ-gliadins, four from LMW glutenin subunits and 16 were from HMW glutenins. The QTOF DIA approach did identify a greater number of longer peptides (79% of peptides comprising 10–24 residues compared to 63% with the LTQ; Figure 4A), many of which represented missed cleavages, with the shorter peptides identified on the LTQ often being derived from the longer peptides identified by the QTOF. The shorter peptides have a lower probability of spanning a variant amino acid position in a given protein sequence, reducing their capacity to support identification of individual isoforms of different gluten proteins compared to the longer peptides.

**Figure 5:**
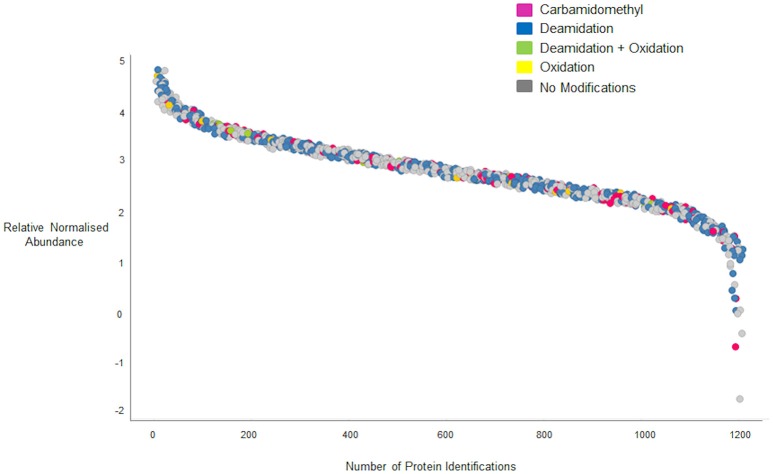
**Quantification curve for all peptides (modified and unmodified) identified on the QTOF**. Gray circles, peptides with no modifications; yellow circles, peptides with oxidized methionine residues; green circles, peptides with deamidated glutamine residues; blue circles, peptides carrying both oxidized methionine and deamidated glutamine residues; pink circles, peptides carrying Carbamidomethyl modifications. Relative normalized abundance is shown on the y axis and the protein index shown on the x-axis.

In the data gathered using the QTOF, a significant increase in the number of peptides identified was observed for peptides of 21 residues in length, with a peak of 106 peptides that does not fit the general distribution curve observed. The dynamic range of intensity for these peptide peaks was within the range observed for all the peptides identified suggesting they did not ionize any differently to the other peptides and may simply reflect the distribution of chymotryptic cleavage sites (Figure S6). A full list of the 21 residue peptides observed can be found in Supplementary Material, Data Sheet 4, with the corresponding protein accession, peptide sequence and modifications. Whilst the majority of peptides identified by both platforms were unmodified (Figures 4C1,C2) deamidation was observed in 40 and 37% of peptides identified respectively by the QTOF and LTQ platforms (Figures 4B,C). In general, deamidation was observed irrespective of peptide length apart from 21 residue peptides, 75% of which were deamidated (Figure 4B). In contrast, oxidation of methionine, which commonly occurs during sample preparation, was identified in ~3% of peptides identified by both platforms. In order to ascertain whether the deamidation was extensive, the relative normalized abundance of the unmodified and modified peptides were defined (Figure 5A). These data indicate that the deamidation was a widespread modification that spanned the range of abundancies observed.

**Figure 6:**
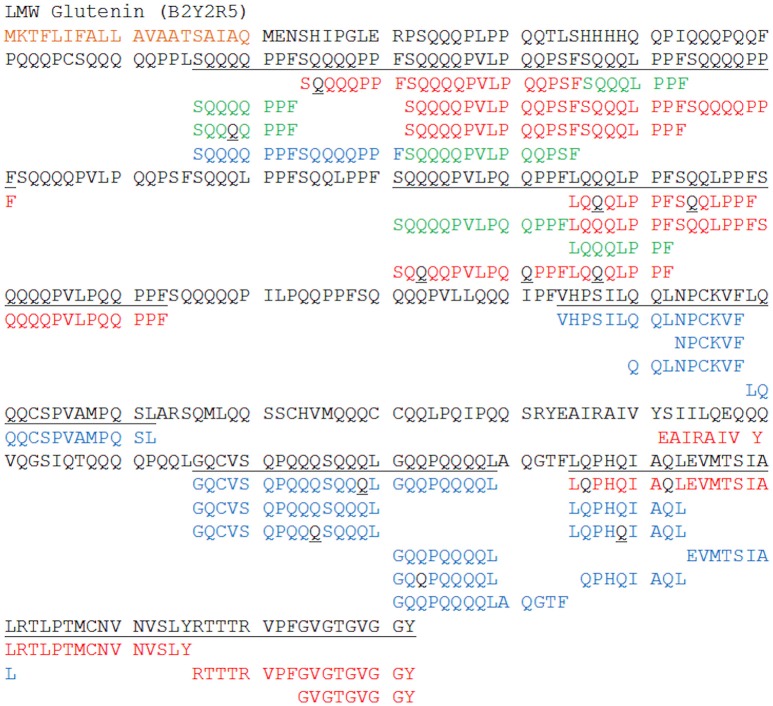
**Sequence coverage of an exemplar LMW glutenin subunit (B2Y2R5)**. Peptide sequences unique to the QTOF with ion mobility incorporated DIA are shown in red, sequences unique to the LTQ with DDA in blue with shared peptides shown in green. Modified amino acids are highlighted in black; with black Q's representing deamidated glutamine. When the residue is black and underlined this represents that the modification could occur at multiple points within the sequence, and its exact location is unknown. The signal peptide is highlighted in orange. The underlined sequence represents the regions of the sequences covered by the peptides from either the QTOF or LTQ data.

The combined data from the QTOF and LTQ platforms enabled vastly improved sequence coverage to be obtained for many protein identifications made, as illustrated in Figure 6 for the LMW glutenin protein, B2Y2R5. The N-terminal sequences of the LMW glutenins are distinctive and have been used to classify this gluten sub-group (Shewry et al., 1986). B2Y2R5 should classically have an N-terminal sequence starting at residue 21 with the sequence ^21^MENSHIPGL^29^, distinctive of a LMW-s glutenin subunit (Shewry et al., 1986). However, no peptide corresponding to this N-terminal sequence was identified; however due to the extensive sequence coverage and four peptides unique to that protein being observed on both platforms it can be confidentially identified. Deamidated residues (highlighted in black in Figure 6) were observed at various positions across the protein, and sometimes at multiple positions in the same peptide. However, in some instances, where there are runs of glutamines, the exact location of the deamidation is ambiguous, and the same *m/z* would be observed for the same peptide with the modification at multiple locations, this is highlighted in Figure 6 when black residues are underlined.

## Discussion

The highly complementary repertoire of peptides identified by the different platforms used in this study enabled a greater number of gluten specific peptides and protein accessions to be identified than have previously been reported using only MS methodology (Mamone et al., 2011; Uvackova et al., 2013; Fiedler et al., 2014; Prandi et al., 2014; Colgrave et al., 2015; Manfredi et al., 2015; Wang et al., 2015; van den Broeck et al., 2015; Barro et al., 2016; Martínez-Esteso et al., 2016) and is comparable with those identified using gel-based separation techniques (Dupont et al., 2011). This approach also increased the sequence coverage that could be achieved using data collected using only one platform, giving greater assurance of the “trueness” of protein identifications made. It has been previously shown that utilizing complementary instruments can dramatically improve the proteome coverage (Elias et al., 2005) and the low level of overlap observed at the peptide level can be explained by fundamental differences between the platforms such as ionization source. Additionally data processing for the QTOF excluded some shorter peptides for the purpose of sequence coverage comparison only. Furthermore, the QTOF platform employed ion mobility, a method that has previously been shown to enhance coverage of the serum proteome by 85% (Daly et al., 2014). Lastly, the different modes of data acquisition used also play a role since in DDA only peptides with signals that rise above the noise in a full-scan MS spectrum are selected for fragmentation (Doerr, 2015), which may represent as little as 16% of the total peptides in a sample (Michalski et al., 2011). In contrast in DIA the full spectrum is acquired and peptides are not fragmenting peptides based on predefined thresholds (Chapman et al., 2014).

More detailed comparison with previous untargeted MS profiling studies is difficult because they have either been undertaken with the aim of identifying peptide targets for detection of gluten (Colgrave et al., 2015; van den Broeck et al., 2015; Martínez-Esteso et al., 2016), or used only tryptic digestion and consequently identified only a few gluten protein accessions (Uvackova et al., 2013). A further complication is that putative identifications have also often been made using protein sequence accessions from other, closely related species such as *Triticum diccocum* and *Aegilops squarrosa* (Martínez-Esteso et al., 2016). Surprisingly, almost exactly the same proteins were profiled in extracts prepared using reducing agent and 75% (v/v) ethanol (E2 and E3) as were identified in an extract containing little immunoreactive gluten. Thus, unexpectedly, sample preparation was not the limiting factor in the capacity to comprehensively profile the gluten proteins. This highlights the highly sensitive nature of the mass spectrometry platforms able to detect proteins that are not optimized for extraction.

Widespread deamidation was observed in all the different types of gluten proteins identified, and observation also made by Martínez-Esteso et al., (2016) in their analysis of a gluten food ingredient. This may be a methodological artifact as, although glutamine is usually much less susceptible to deamidation than asparagine under neutral and alkaline conditions (Robinson, 2002) acidic conditions appear to favor the deamidation of glutamine residues (Joshi and Kirsch, 2002), even though the only acidic environment experienced by the proteins for a prolonged period of time is exposure to dilute formic acid solutions. It is also possible that deamidation occurs naturally in the plant, since trains of spots of the same Mr and different pI are frequently observed in 2D PAGE profiles of gluten. Although these are often attributed to sample preparation procedures (Johnson et al., 2016) they are present in protein from freshly isolated protein bodies which has not been exposed to extreme conditions before analysis (Field et al., 1982). Further studies modifying the extraction methods could be used to identify whether the deamidation of gluten is an artifact of the analysis or a genuine, previously unidentified, post-translational modification or an artifact of the extraction and analytical procedures. Anomalous N-terminal proteolytic processing of a LWM subunit was also observed, with a long peptide identified which spans the signal peptidase cleavage site to give the well-defined N-terminal consensus motif METSRV. Ragged N-terminal processing is not uncommon in plant seed proteins and has been described in purified plant proteins, such as the 2S albumins from Brazil nut (Moreno et al., 2004) and there is some variation in further proteolytic processing of gluten protein's by a putative vascular asparaginyl endoprotease (Egidi et al., 2014).

The low content of arginine and lysine residues in gluten proteins means that protease digestion must be carried out using less-commonly used and less predictable enzymes such as chymotrypsin. However, the distribution of chymotryptic cleavage sites in many proteins means this enzyme typically generates longer peptides than are generally encountered when digesting proteins with trypsin. These longer peptides may present a challenge for data acquisition using MS. The capacity to sequence longer peptides in the current study, especially by the QTOF platform (often resulting from missed cleavages) also enabled greater sequence coverage and supported greater specificity of protein identification and the individual isoform level. This was also observed when profiling peanut allergens (Johnson et al., 2016) and hence whilst there have been many concerns about efficiency of protease digestion steps, which maybe crucial for effective quantification, for identification purposes missed cleavages may enhance levels of identification when using platforms able to sequence longer peptides.

The HMW subunit Dx3 is usually considered to be a “mutant” form closely related to Dx2, due to the similar mobility observed on the SDS-PAGE. However, this has not been confirmed by direct analysis at the gene or protein level. The identification here of sequences corresponding to the established sequences of HMW subunits Dx2 and Dx5 therefore casts doubt on this assumption and suggests subunit Dx3 may in fact contain sequences similar to both the Dx2 and Dx5. This is supported by ESIMS analysis of the Dx2 and Dx3 subunits that showed signification differences in their Mr (Lagrain et al., 2013). Furthermore, when subunits Dx2, Dx3, and Dx5 are shown on SDS-PAGE the bands are not distinguishable (Lagrain et al., 2012), giving rise to the need to further clarify the Dx3 subunit through sequencing this protein. The presence of these sequences attributed to the unexpected HMW glutenin could go some way to explain why Hereward has unexpected good bread making quality.

Our data show that comprehensive proteomic profiling of plant proteins, such as gluten, is not limited by the proteomics methodology, but by access to appropriate genetic data in a form usable for proteomic informatics pipelines which can handle highly polymorphic proteins with regards amino acid substitutions, which have both repeating sequences and deletions (Vensel et al., 2011; Kasarda et al., 2013; Romero-Rodríguez et al., 2014). Such integrated approaches will be required to unravel why certain wheat cultivars, such as Hereward, have better than expected bread making quality and hence identify novel targets for crop improvement. These data will also facilitate the development of future approaches well-founded targeted MS methods of analysis of gluten in foods.

## Author contributions

SB, LG, JL, PS, MJB, MB, and EM all gave substantial contributions to the concept and design of the work, and where involved in the acquisition, analysis and interpretation of data at varying points. All authors were involved in the drafting of the manuscript, and gave approval prior to submission. All authors acknowledge accountability for all aspects of the work.

### Conflict of interest statement

LG and JL declare a financial relationship that could be perceived to influence or give the appearance of potentially influencing the work submitted as both are employed by the one of the mass spectrometry vendors reviewed in this paper. MJB declares a potential conflict of interest as he works within a consulting capacity for Synergy Health Plc, who carry out analysis to detect and quantify gluten in food products. The other authors declare that the research was conducted in the absence of any commercial or financial relationships that could be construed as a potential conflict of interest.

## Abbreviations

ATEE: N–Acetyl–L–tyrosine ethyl ester
BCIP: 5-bromo-4-chloro-3′-indolyphosphate p-toluidine salt
CCD: Charge coupled device
CD: Coeliac disease
cv.: Cultivar
DDA: Data dependent acquisition
DIA: Data independent acquisition
DIA-IM-MS: Data independent acquisition with incorporated ion mobility mass spectrometry
EIC: Extracted Ion Chromatogram
ELISA: Enzyme linked immunosorbent assay
FDR: False discovery rate
HMW: High molecular weight
IEF: Isoelectric focusing
LDS: Lithium Dodecyl Sulphate
LTQ: Linear ion trap
LMW: Low molecular weight
mAbs: Monoclonal Antibody
Mr: Molecular Mass
NBT: Nitro-blue tetrazolium chloride
QTOF: Quadrupole time of flight
T3PQ: Top 3 protein quantification
Tris: Tris (hydroxymethyl) aminomethane.
