# Supplementary material for: Comprehensive Proteomic Profiling of Wheat Gluten Using a Combination of Data-Independent and Data-Dependent Acquisition
Source: Front Plant Sci. 2017 Jan 10;7:2020. doi: 10.3389/fpls.2016.02020 (PMC5223596; doi:10.3389/fpls.2016.02020)
Supplement: Supplementary file 1 [file Table1.DOCX]

*Supplementary Table S1: Sequence comparison of HMW subunits identified by proteomic profiling using minimum of 3 peptides. Sequence comparison was undertaken by Clustal Omega using a sequence identity calculation, which accounts for insertions and deletions within the sequence. The sequence in bold is the master sequence and related to the expected subunits present in Wheat cv Hereward. For the non-master sequences their corresponding subunits appear in brackets after the accession number. An asterisk is used when the origin of the subunit is not verifiably known. The number of unique peptides for each protein is also shown.*

| **HMW Subunit type** | **Sequence Accession No** | **% Sequence identity** | **Number of unique peptides** |
| --- | --- | --- | --- |
| **Dx2**  **P08489**  [Master Sequence] | D0IQ05 (1Dx5) | 94 | 8 |
|  | Q599I0 (1Dx2.2) | 81.8 | 8 |
|  | A0MZ38 (1Ax) | 68.8 | 13 |
| 1Bx7  **Q42451**  [master sequence] | Q6UKZ5 (1Bx14) | 93.9 | 20 |
|  | Q1KL95 (1Bx*) | 99.1 | 6 |
|  | A5HMG1 (1Bx13) | 96.6 | 5 |
|  | M4M8L5 (1Bx17) | 90.1 | 3 |
|  | Q6Q7J1 (1Bx23) | 96.7 | 13 |
