# Supplementary figures and images for: Comprehensive Proteomic Profiling of Wheat Gluten Using a Combination of Data-Independent and Data-Dependent Acquisition
Source: Front Plant Sci. 2017 Jan 10;7:2020. doi: 10.3389/fpls.2016.02020 (PMC5223596; doi:10.3389/fpls.2016.02020)

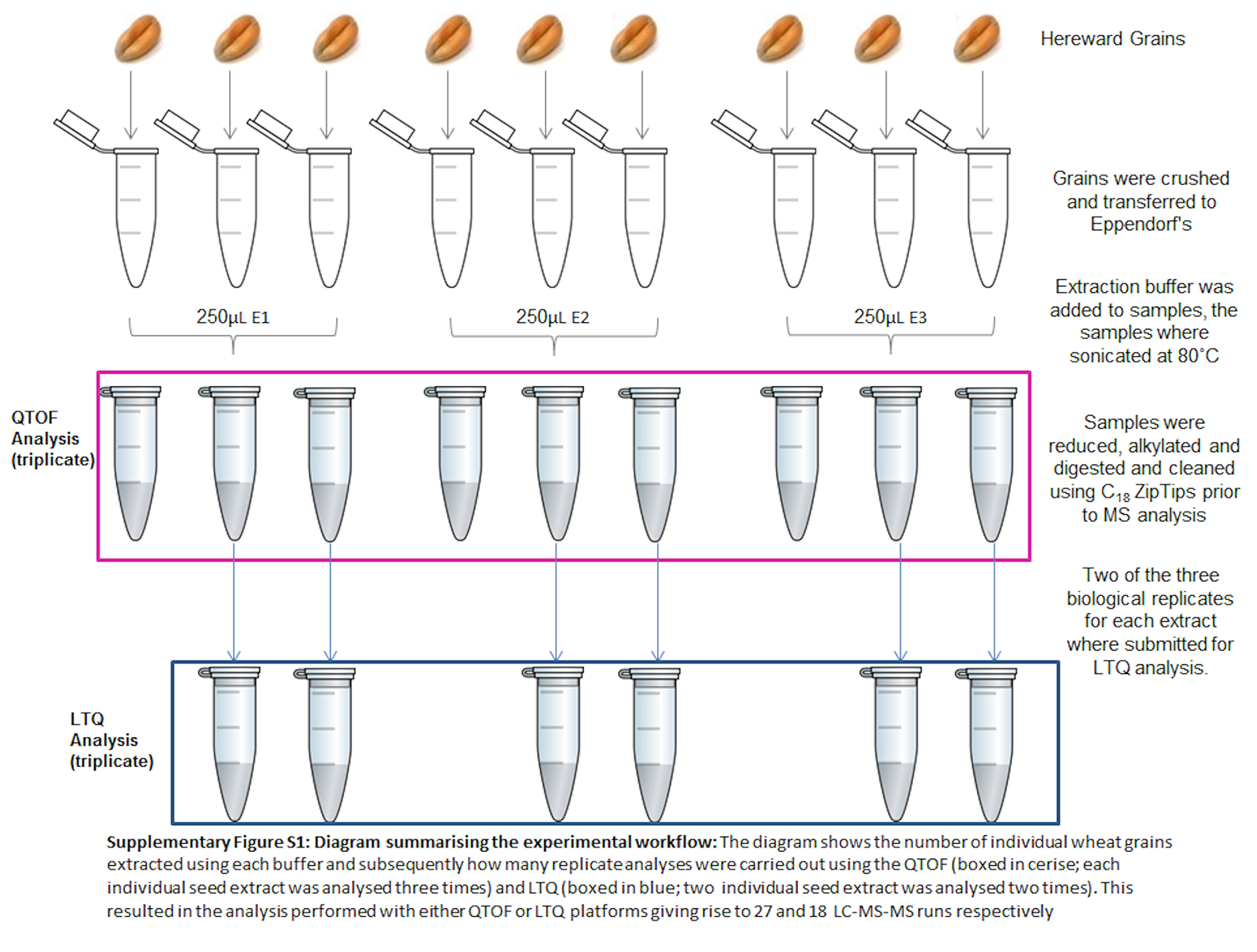

Supplement: Supplementary file 6 [file Image1.tif]

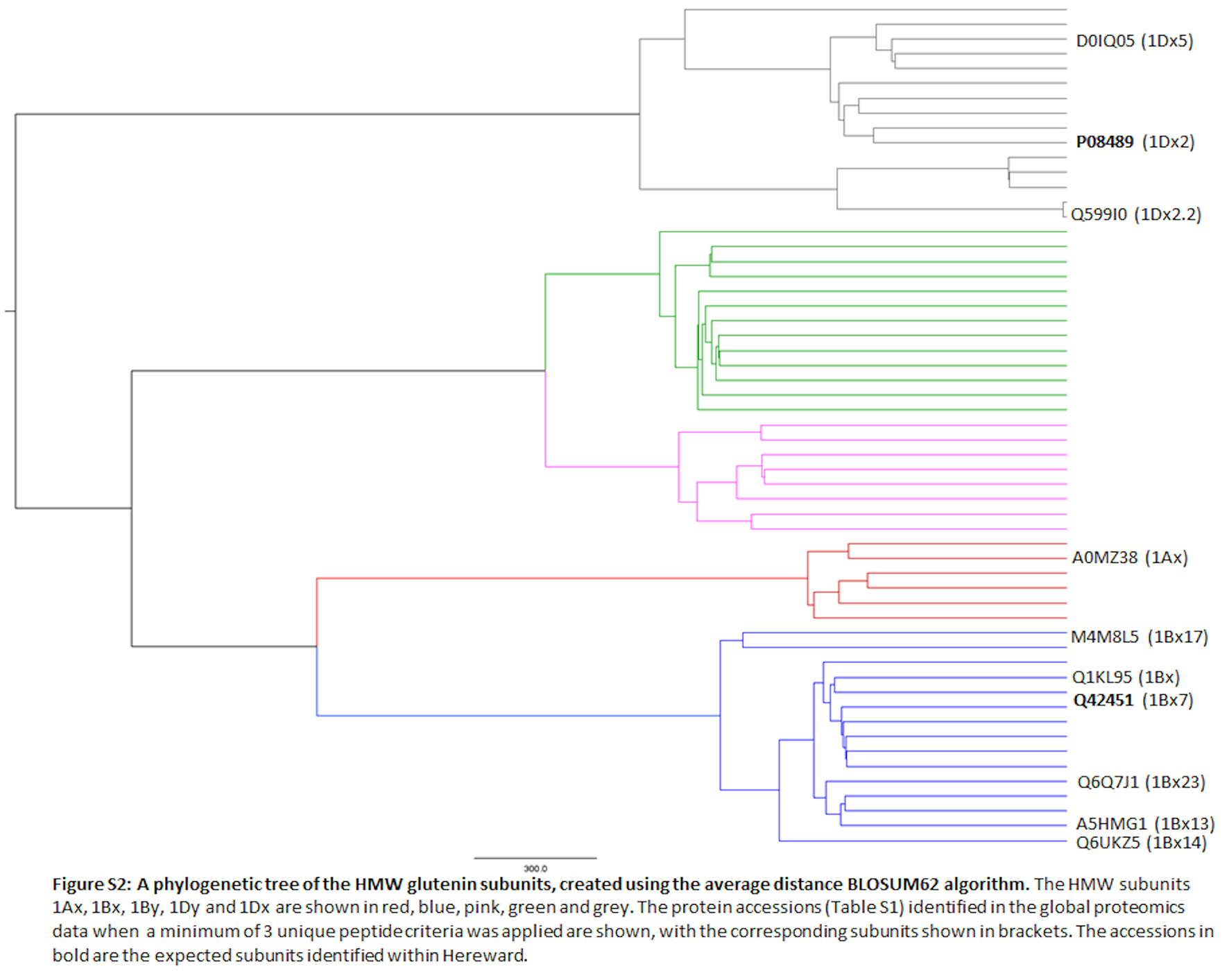

Supplement: Supplementary file 7 [file Image2.TIF]

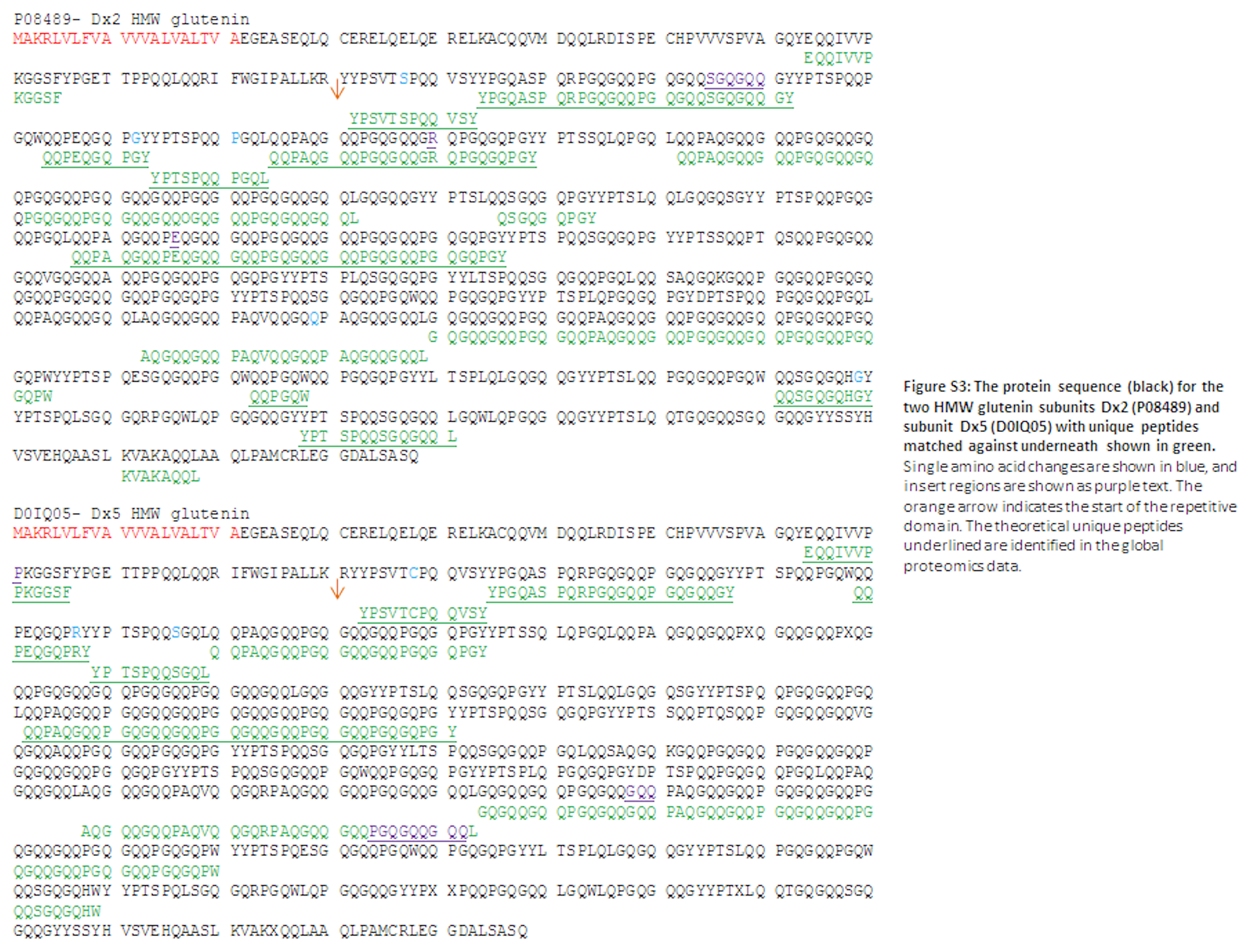

Supplement: Supplementary file 8 [file Image3.TIF]

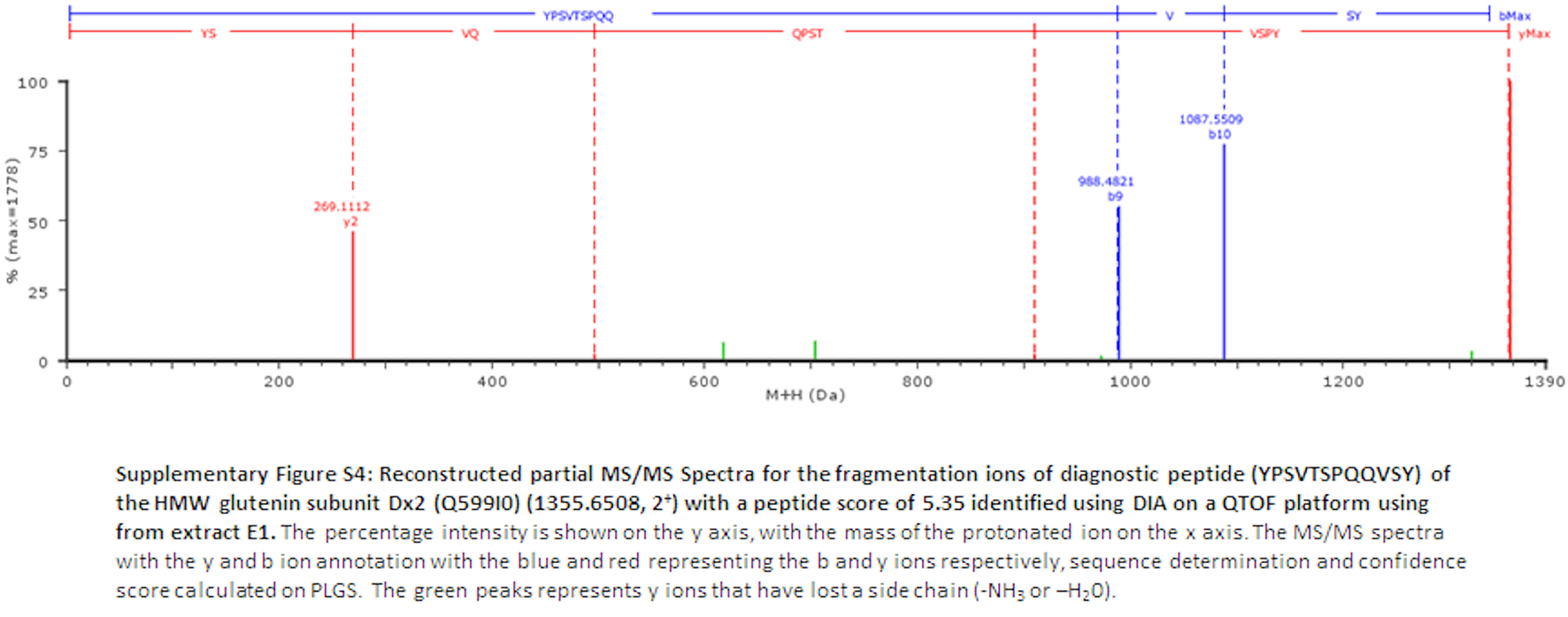

Supplement: Supplementary file 9 [file Image4.TIF]

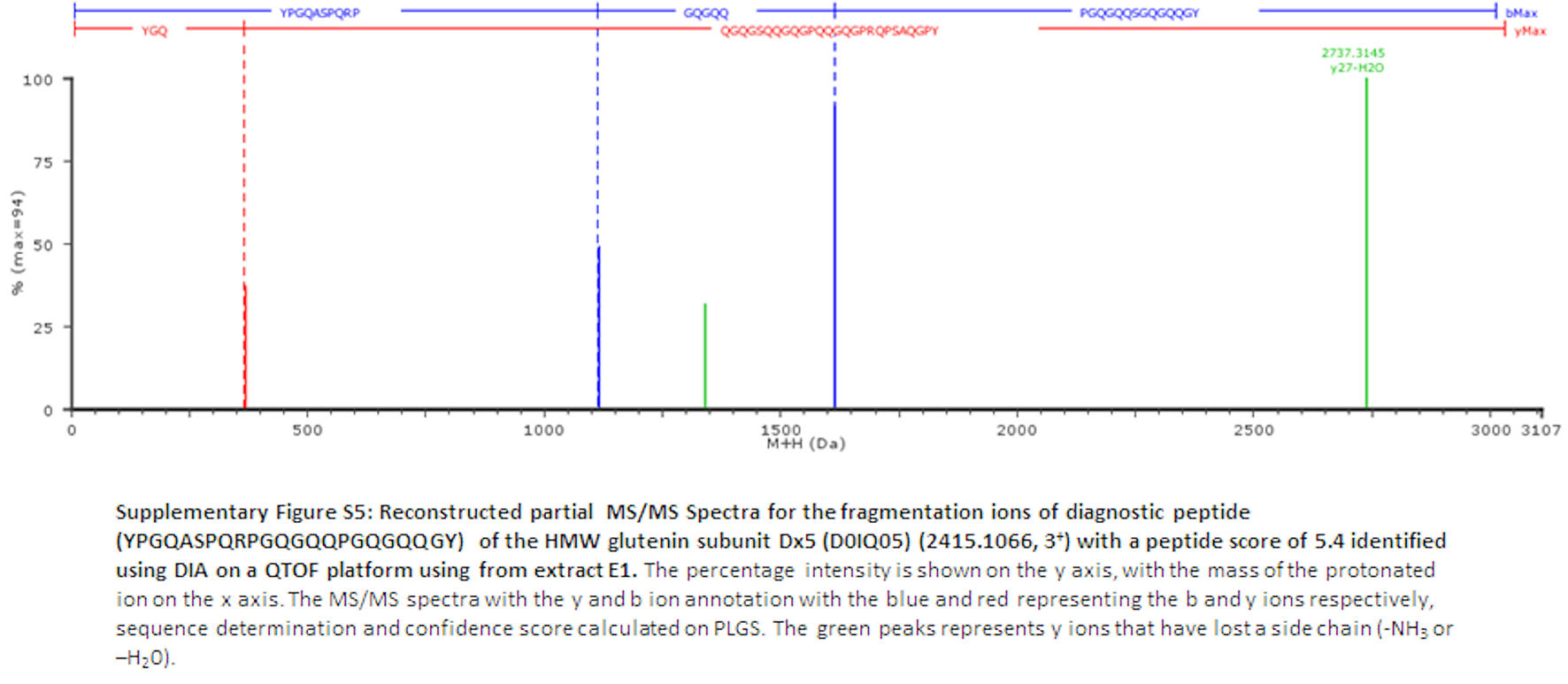

Supplement: Supplementary file 10 [file Image5.TIF]

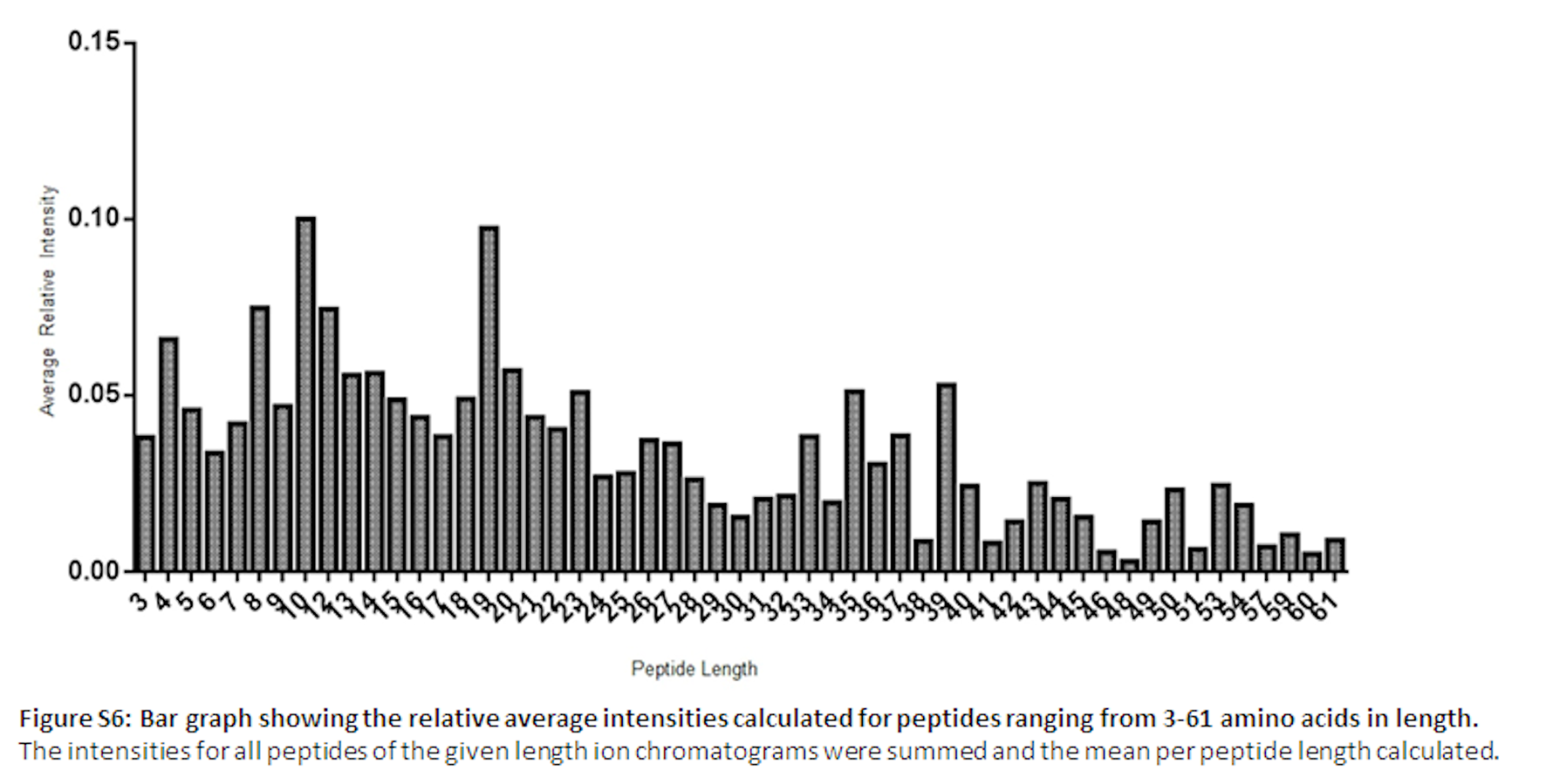

Supplement: Supplementary file 11 [file Image6.TIF]

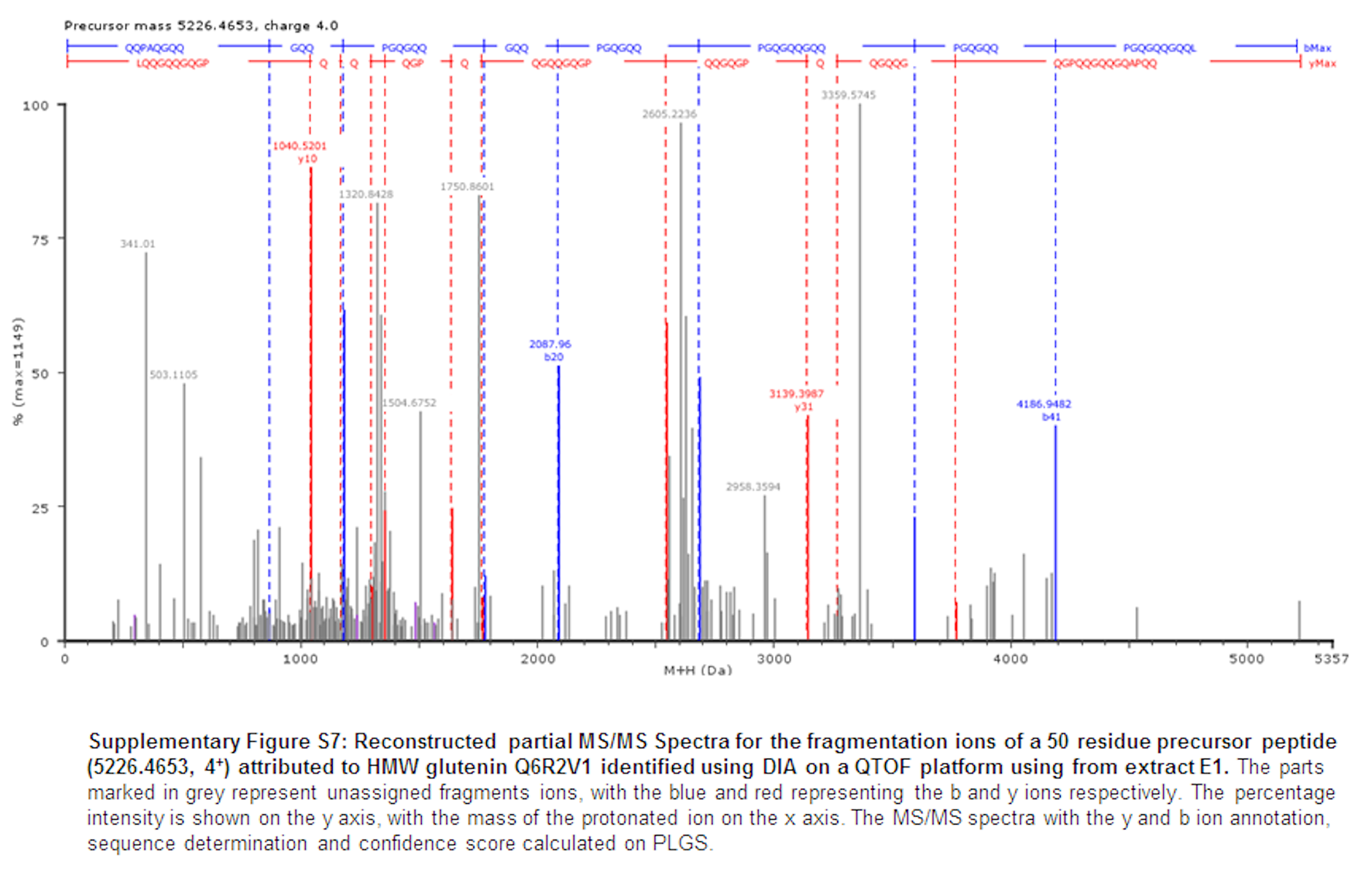

Supplement: Supplementary file 12 [file Image7.TIF]
